# Meta-analysis of secondary hematologic malignancy risks following radioactive iodine therapy for differentiated thyroid cancer

**DOI:** 10.1097/JS9.0000000000002813

**Published:** 2025-06-24

**Authors:** Hongyue Su, Fan Yang, Zhiyuan Wang, Xiaoyu Ji, Liang Shao, Lili Zhu, Hao Zhang, Wenqian Zhang, Wei Sun

**Affiliations:** a Department of Thyroid Surgery, The First Hospital of China Medical University, Shenyang, Liaoning, China; b Department of Dermatology, The People’s Hospital of Liaoning Province, Shenyang, Liaoning, China; c Department of Head and Neck Surgery, Cancer Hospital of China Medical University, Shenyang, Liaoning, China

**Keywords:** differentiated thyroid cancer, hematologic malignancies, meta-analysis, radioiodine therapy, second primary malignancy

## Abstract

**Purpose:**

Radioactive iodine (RAI) therapy remains a widely accepted and effective intervention for differentiated thyroid cancer (DTC). Nonetheless, concerns persist regarding the potential emergence of second primary malignancies following RAI exposure. To assess the association between RAI administration and the risk of secondary hematologic malignancies in DTC patients, a meta-analysis was conducted.

**Methods:**

A systematic literature search was performed via PubMed and Web of Science, conforming to PRISMA guidelines.

**Results:**

Seventeen studies, including a total of 586 260 DTC patients, were included in the final analysis. The aggregated risk ratio (RR) for secondary hematologic malignancies among those treated with RAI was 1.25 (95% CI: 1.13–1.38, *P<*0.0001), compared with non-RAI recipients. Heterogeneity across studies was minimal (*I*^2^ = 8%). Further subgroup analyses were conducted based on malignancy subtype and RAI dosage. The RR estimates by subtype were as follows: non-Hodgkin’s lymphoma, 0.91 (95% CI: 0.72–1.16, *P* = 0.45); Hodgkin’s lymphoma, 1.17 (95% CI: 0.62–2.21, *P* = 0.63); myeloma, 0.90 (95% CI: 0.59–1.37, *P* = 0.62); and leukemia, 1.52 (95% CI: 1.05–2.21, *P* = 0.03). For patients receiving RAI doses below 3.7 GBq, the pooled RR was 0.39 (95% CI: 0.22–0.72, *P* = 0.002). Sensitivity and subgroup analyses reinforced the robustness and internal consistency of these findings. No publication bias was detected via Egger’s regression or Begg’s funnel plot (*P =* 0.511).

**Conclusion:**

These results suggest a potential elevation in the risk of secondary hematologic malignancies – particularly leukemia – following RAI therapy in DTC patients. However, administration of RAI at doses below 3.7 GBq may not confer such risk. Additional large-scale, multicenter prospective investigations are warranted to validate these observations.

## Introduction

The global incidence of thyroid cancer has markedly risen over recent decades^[1]^. Data from the National Cancer Center indicate that thyroid cancer ranks third in incidence nationwide, occupying the seventh position among newly diagnosed cancers in males and third in females^[2]^. Papillary carcinoma represents the predominant histological subtype, followed by follicular carcinoma; together, they account for over 90% of all thyroid malignancies. Total thyroidectomy remains the standard therapeutic approach for differentiated thyroid cancer (DTC), as recommended by the American Thyroid Association (ATA) Management Guidelines. The administration of radioactive iodine (RAI) therapy is contingent upon histopathological assessment and the presence of residual, unresectable, or metastatic lesions^[3]^.

**HIGHLIGHTS**

- First use of 17 studies to demonstrate the relationship between RAI and SHMS through meta-analysis
- DTC patients receiving RAI treatment could have a higher risk of developing SHMS
- When the dose of RAI is less than 3.7 GBQ, it may be safe and will not cause SHMS

Iodine accumulates at markedly elevated concentrations in the thyroid gland through a carrier-mediated mechanism; however, comparable uptake of ^131^I has also been identified in other organs, including salivary and lacrimal glands. Substantial evidence indicates that RAI exposure damages these glands, contributing to the onset of Sicca syndrome^[4]^. Moreover, concerns persist regarding the potential association between RAI therapy and the emergence of second primary malignancies (SPMs). As the majority of DTC survivors experience extended survival, comprehensive risk assessment of RAI-induced SPMs within this population is essential. Although the ATA guidelines acknowledge this issue, the relationship between postoperative RAI therapy and SPM development in thyroid cancer patients remains inconclusive and warrants further robust investigation.

The development of second hematologic malignancies (SHMs) following initial cancer treatment is a rare yet devastating clinical outcome. Historical data from atomic bomb survivors have established radiation exposure as a risk factor for leukemia, exhibiting a dose-dependent linear relationship^[5]^. Consequently, the potential leukemogenic effect of RAI warrants further scrutiny. In a large-scale study by Molenaar *et al*, 47% of 148 215 DTC patients received RAI, while 53% underwent surgery alone. Over a median follow-up of 6.5 years, 783 cases of SHMs were identified, with an increased early incidence of acute myeloid leukemia and chronic myeloid leukemia observed exclusively in the RAI-treated cohort; no other hematologic malignancies were reported. Furthermore, RAI-associated acute myeloid leukemia exhibited a poor clinical outcome^[6]^. Complementary findings from a nationwide Korean cohort indicated a significant link between cumulative RAI doses exceeding 3.7 GBq and subsequent leukemia onset^[7]^. Despite these observations, a formal petition published on 26 March 2018, by Mark *et al*, and endorsed by 67 nuclear medicine specialists from 16 countries, challenged the conclusions of Molenaar *et al*, citing methodological bias and the omission of evidence suggesting RAI’s potential protective role against certain cancers^[8]^. Moreover, several investigations have reported no significant association between RAI administration or dosage and the risk of SHMs^[9,10]^.

Owing to limited sample sizes in prior studies, the association between RAI and SHMs has remained unclear. The ATA guidelines similarly classify the evidence as low quality and issue only tentative recommendations regarding this link^[3]^. This study aims to assess the potential risk of SHMs in DTC patients treated with RAI and to investigate the possible associations with RAI subtypes and administered doses. All aspects of the study adhered to the TITAN Guidelines 2025^[11]^.

## Methods

### Search strategy and study selection

Conducted in alignment with the PRISMA guidelines (Preferred Reporting Items for Systematic Reviews and Meta-Analyses)^[12]^, this meta-analysis employed a systematic search strategy using PubMed and Web of Science to identify relevant studies published up to 10 October 2024. The search incorporated predefined keywords to ensure comprehensive and targeted literature retrieval: “differentiated thyroid cancer” [All fields] OR “DT” [All fields] OR “cancer” [All fields] OR “thyroid carcinoma” [All fields] OR “thyroid neoplasm” [All fields] OR “papillary thyroid carcinoma” [All fields] OR “follicular thyroid carcinoma” [All fields] AND “Hematologi” [All fields] OR “leukemi” [All fields] OR “lymphom” [All fields] OR “myelom” [All fields] AND “second primary malignancy” [All fields] AND “radioiodine therapy” [All fields] OR “RAI therapy” [All fields] OR “RAI therapy” [All fields].

To ensure alignment with the intended predictors, studies were included only if all of the following criteria were satisfied: (1) randomized or nonrandomized controlled trials, or prospective or retrospective studies were employed; (2) risk assessments of SHMs in DTC patients treated with or without RAI therapy were reported; (3) DTC diagnosis was pathologically confirmed either intraoperatively or postoperatively; (4) SHM presence was validated through pathological evaluation; and (5) sufficient data were available to calculate the risk ratio (RR) and corresponding 95% confidence interval (CI). Studies were excluded if they were review articles, conference abstracts, correspondence, editorials, or lacked clinicopathologic data necessary for meta-analysis.

The formulation of the research question guided the initial title and abstract screening, independently conducted by two reviewers. Full-text screening was conducted for articles retained after preliminary evaluation. Discrepancies were resolved through discussion or, when consensus was not achieved, adjudicated by a third reviewer.

### Data extraction

Data extracted from each eligible article included: first author, publication year, country of origin, diagnostic classification, total patient cohort size, patient distribution by RAI dose (<3.7 GBq vs >3.7 GBq), incidence of Hodgkin’s lymphoma, non-Hodgkin’s lymphoma, myeloma, and leukemia, follow-up duration, and methodological quality score.

### Quality evaluation

The methodological quality of the case-control studies was assessed using the Newcastle-Ottawa Scale (NOS) by two independent reviewers, yielding total scores ranging from 0 to 9^[13]^. Discrepancies were resolved through consensus. The NOS is widely applied in observational research, including cohort and case-control designs, and appraises studies based on three domains: selection of study participants (4 items), comparability of groups (2 items), and assessment of either exposure or outcome (3 items). Studies achieving full scores in selection (4 points), comparability (2 points), and outcome/exposure assessment (3 points) were categorized as having a low risk of bias. High-risk bias was assigned to studies scoring 1 or 0 in selection, 0 in comparability, or 0 in exposure/outcome assessment. Intermediate scores indicated a moderate risk of bias. All studies classified as moderate risk were included in the current review^[14,15]^.

### Pooling of data and statistics

RR and corresponding 95% CIs were calculated to quantify the associations. Heterogeneity was assessed using both the *I*^2^ statistic and the χ^2^-based Q test^[16]^. When heterogeneity was not detected (*P >* 0.1, *I*^2^ < 50%), a fixed-effect model was applied. In contrast, the presence of heterogeneity (*P<* 0.1, *I*^2^ > 50%) warranted the use of a random-effects model based on the DerSimonian and Laird approach. A pooled RR and its 95% CI were derived accordingly. An RR exceeding 1 indicated an increased risk^[17]^. The choice of analytical model influences interpretation: the fixed-effect model assumes a single true effect size shared across all studies, whereas the random-effects model incorporates the possibility of variability in true effect sizes across populations. Under the fixed-effect model, the summary estimate reflects the common effect but does not permit evaluation of between-study variance. In contrast, the random-effects model generates a summary estimate representing the mean of the distribution of effects and enables assessment of effect size variability across populations^[18]^.

Subgroup analyses were stratified by hematological disease subtype and administered RAI dose. To evaluate the robustness of the pooled estimates, a sensitivity analysis was conducted by sequential exclusion of individual studies, determining the extent to which any single large or outlier study influenced overall results. Publication bias was assessed through Begg’s funnel plot, and further quantified using Egger’s linear regression test, with statistical significance set at *P<* 0.1^[19,20]^.

Statistical computations were performed using Review Manager 5.4 (Cochrane Collaboration, Northern Europe) and Stata 13.0 (Stata Corp LP, College Station, Texas) for sensitivity assessments. Unless otherwise specified, *P*-values < 0.05 were interpreted as indicative of statistical significance.

This study adhered to PRISMA (Preferred Reporting Items for Systematic Reviews and Meta-Analyses) and AMSTAR (Assessing the Methodological Quality of Systematic Reviews) guidelines throughout its reporting process^[21]^.

## Results

### Study selection

**Figure 1.**
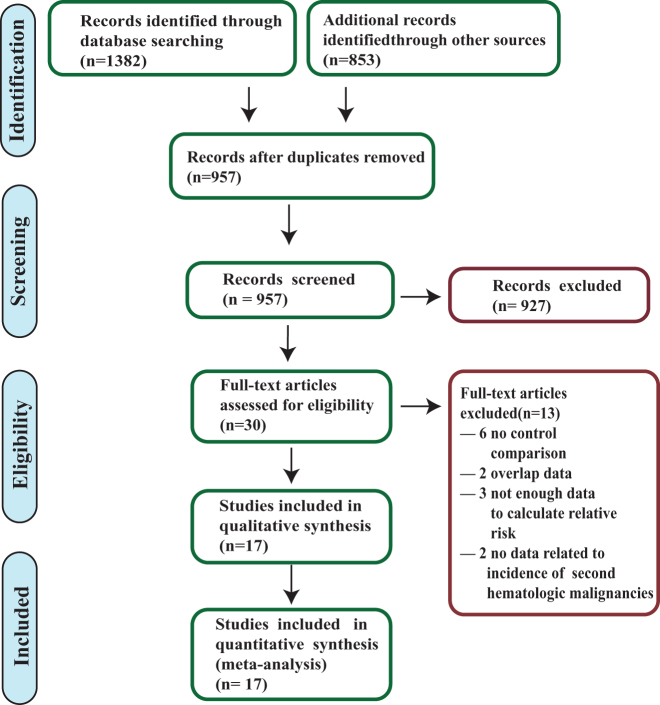
Study selection process illustrated via PRISMA flow diagram.

Figure 1 summarized the systematic search process. The initial database screening yielded 2235 potentially relevant citations. Following the exclusion of 1278 duplicates, 957 unique records were identified. Title and abstract screening led to the removal of 927 citations. Full-text assessment of the remaining 30 articles resulted in the exclusion of 13 studies. The final set of 17 eligible articles^[6,7,9,22–35]^ included 586 260 patients. Of these, 281 585 received postoperative RAI, while 304 675 did not, forming the comparative basis for the present meta-analysis.

### Analysis and characteristics of the selected articles

Table 1 presented the demographic and clinical profiles of patients incorporated into the meta-analysis. Extracted variables include the first author’s name, publication year, country, diagnostic classification, distribution of patients receiving RAI doses above and below 3.7 GBq, incidence of Hodgkin’s lymphoma, non-Hodgkin’s lymphoma, myeloma, leukemia, duration of follow-up, and assigned quality scores.

**Table 1:** Demographic data and clinical characteristics of overall study population

Study	Year	Country	Diagnosis	Patients (*n*)	Number of patients with RAI >3.7(GBq)	Number of patients with RAI<3.7(GBq)	Hodgkin’s lymphoma	Non-Hodgkin’s lymphoma	Myeloma	Leukemia	Follow-up(years)	Quality Score
C Rubino *et al*	2003	France *et al*	DTC	6841	4	3			6	18	13	8
X Mei *et al*	2021	China	DTC	104 026	-	-				161	9.4	8
RJ Molenaar *et al*	2018	Netherlands	DTC	148 215	-	-					20	8
V Cappagli *et al*	2020	Italy	DTC	1096	-	-					10.52 ± 7.69	8
N Bhattacharyya *et al*	2006	Boston	DTC	29 231	-	-	13		18		4.9	8
BH Lang *et al*	2012	China	DTC	1122	-	-		3			7.8	7
D Hirsch *et al*	2016	Israel	DTC	1943	-	-					9.3	8
M Silva-Vieira *et al*	2017	Portugal	DTC	2031	-	-			2	5	8.8	8
TT Hakala *et al*	2016	Finland	DTC	910	-	-		11			16.2	7
KY Ko *et al*	2015	China	DTC	1834	-	-					5.40 ± 3.36	7
GH Seo *et al*	2015	Korea	DTC	211 360	36	10				72	2.4	9
AP Brown *et al*	2008	U.S.A.	DTC	2158	-	-	24	99	55	82	8.6	8
GH Seo *et al*	2021	Korea	DTC	9548	3	1					11	8
FD Vathaire *et al*	1997	Swedish *et al*	DTC	1771	0	4			2		10	8
E Pasqual *et al*	2022	U.S.A.	DTC	27 050	-	-	10	82	11	43	15	7
KH Al-Qahtani *et al*	2015	Saudi Arabia	DTC	823	-	-					8.05	7
M Endo *et al*	2018	U.S.A.	PTC	3200	-	-	9	112	38	84	12	7

The NOS-based quality evaluations for all included studies were summarized in Table 2. An overall low risk of bias was identified. However, the domains “additional factor” and “representativeness of the cases” demonstrated unclear risk, likely attributable to inconsistent assessment of potential confounders and possible selection bias in patient inclusion.

**Table 2:** Quality assessment of included case–control studies (Newcastle–Ottawa scale)

Study	Selection					Comparability			Exposure				Final score
	Case definition	Representativeness	Control selection	Control definition		Main factor	Additional factor		Ascertainment	Same method of ascertainment for cases and controls	Non-response rate		
C Rubino *et al*	**+**	-	**+**	**+**		**+**	**+**		**+**	**+**	**+**		**8**
X Mei *et al*	**+**	-	**+**	**+**		**+**	**+**		**+**	**+**	**+**		**8**
RJ Molenaar *et al*	**+**	**+**	**+**	**+**		**+**	-		**+**	**+**	**+**		**8**
V Cappagli *et al*	**+**	**+**	**+**	**+**		**+**	-		**+**	**+**	**+**		**8**
N Bhattacharyya *et al*	**+**	**+**	**+**	**+**		**+**	-		**+**	**+**	**+**		**8**
BH Lang *et al*	**+**	-	**+**	**+**		**+**	-		**+**	**+**	**+**		**7**
D Hirsch *et al*	**+**	-	**+**	**+**		**+**	**+**		**+**	**+**	**+**		**8**
M Silva-Vieira *et al*	**+**	-	**+**	**+**		**+**	**+**		**+**	**+**	**+**		**8**
TT Hakala *et al*	**+**	-	**+**	**+**		**+**	-		**+**	**+**	**+**		**7**
KY Ko *et al*	**+**	-	**+**	**+**		**+**	-		**+**	**+**	**+**		**7**
GH Seo *et al*	**+**	**+**	**+**	**+**		**+**	**+**		**+**	**+**	**+**		**9**
AP Brown *et al*	**+**	**+**	**+**	**+**		**+**	-		**+**	**+**	**+**		**8**
GH Seo *et al*	**+**	**+**	**+**	**+**		**+**	-		**+**	**+**	**+**		**8**
FD Vathaire *et al*	**+**	**+**	**+**	**+**		**+**	-		**+**	**+**	**+**		**8**
E Pasqual *et al*	**+**	-	**+**	**+**		**+**	-		**+**	**+**	**+**		**7**
KH Al-Qahtani *et al*	**+**	-	**+**	**+**		**+**	-		**+**	**+**	**+**		**7**
M Endo *et al*	**+**	-	**+**	**+**		**+**	-		**+**	**+**	**+**		**7**

### Risk of SHMS in patients with DTC receiving RAI

**Figure 2.**
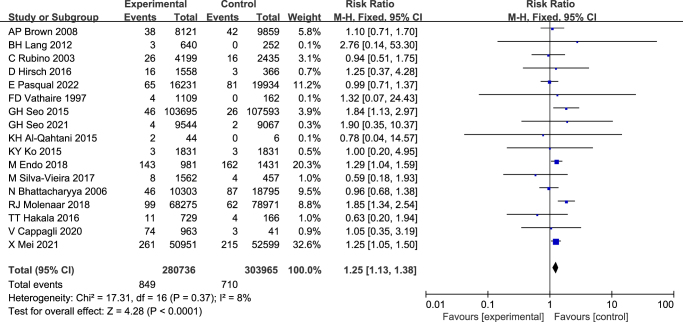
Forest plot summarizing the RR of SHMs in DTC patients treated with RAI compared to untreated counterparts.

**Figure 3.**
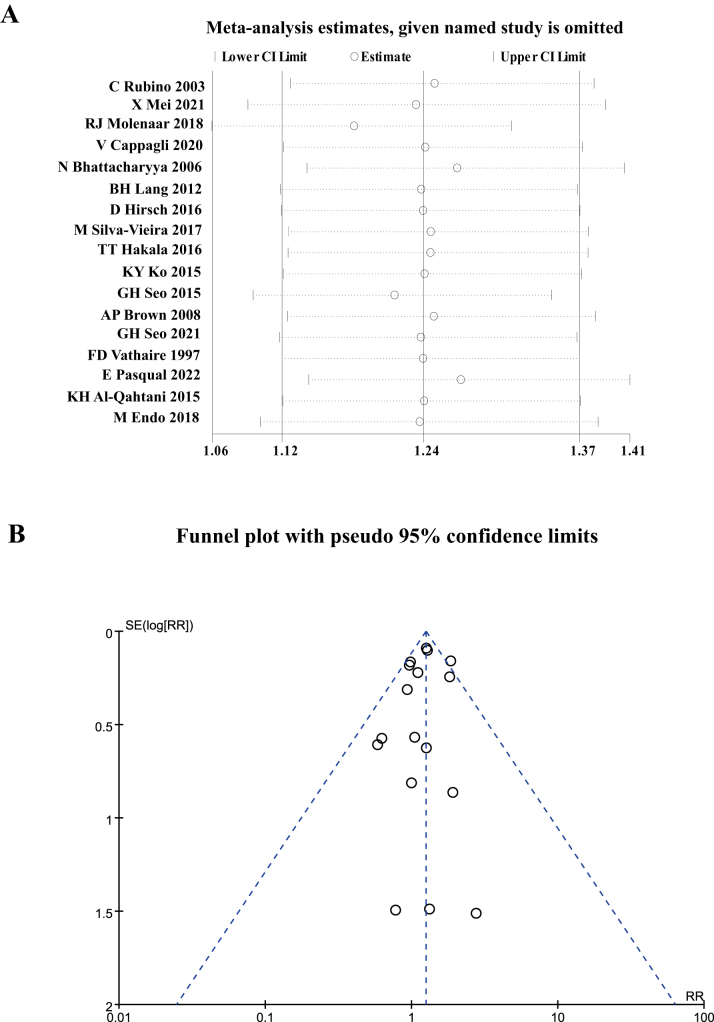
(A) Sensitivity analysis; (B) Funnel plot for the RR of SHMs in DTC patients receiving RAI versus those not exposed.

The analysis employed a fixed-effects model (*P* = 0.37, *I*^2^ = 8%). RAI exposure was significantly associated with an elevated incidence of SHMs (RR = 1.25, 95% CI: 1.13–1.38, *P<* 0.0001) (Fig. 2). Egger’s test and visual inspection of the funnel plot for the fixed-effects model revealed no substantial evidence of publication bias (t = − 0.67; *P =* 0.511) (Fig. 3). Leave-one-out sensitivity analysis confirmed the robustness of the findings, as exclusion of any individual study did not materially alter the overall effect estimate (Table 3). Collectively, these results suggest a 1.25-fold higher risk of SHMs in DTC patients treated with RAI compared to those who did not receive RAI.

**Table 3:** Leave one-out sensitivity analysis

Study omitted	Estimate	[95% Conf. Interval]
C Rubino 2003	1.25	1.13–1.38
X Mei 2021	1.23	1.09–1.39
RJ Molenaar 2018	1.18	1.06–1.31
V Cappagli 2020	1.24	1.12–1.37
N Bhattacharyya 2006	1.27	1.14–1.41
BH Lang 2012	1.24	1.12–1.37
D Hirsch 2016	1.24	1.12–1.37
M Silva-Vieira 2017	1.25	1.12–1.38
TT Hakala 2016	1.25	1.12–1.38
KY Ko 2015	1.24	1.12–1.37
GH Seo 2015	1.21	1.10–1.35
AP Brown 2008	1.25	1.12–1.38
GH Seo 2021	1.24	1.12–1.37
FD Vathaire 1997	1.24	1.12–1.37
E Pasqual 2022	1.27	1.14–1.41
KH Al-Qahtani 2015	1.24	1.12–1.37
M Endo 2018	1.24	1.10–1.39
Combined	1.24	1.12–1.37

### Risk of SHMS in subtypes of hematological diseases

**Figure 4.**
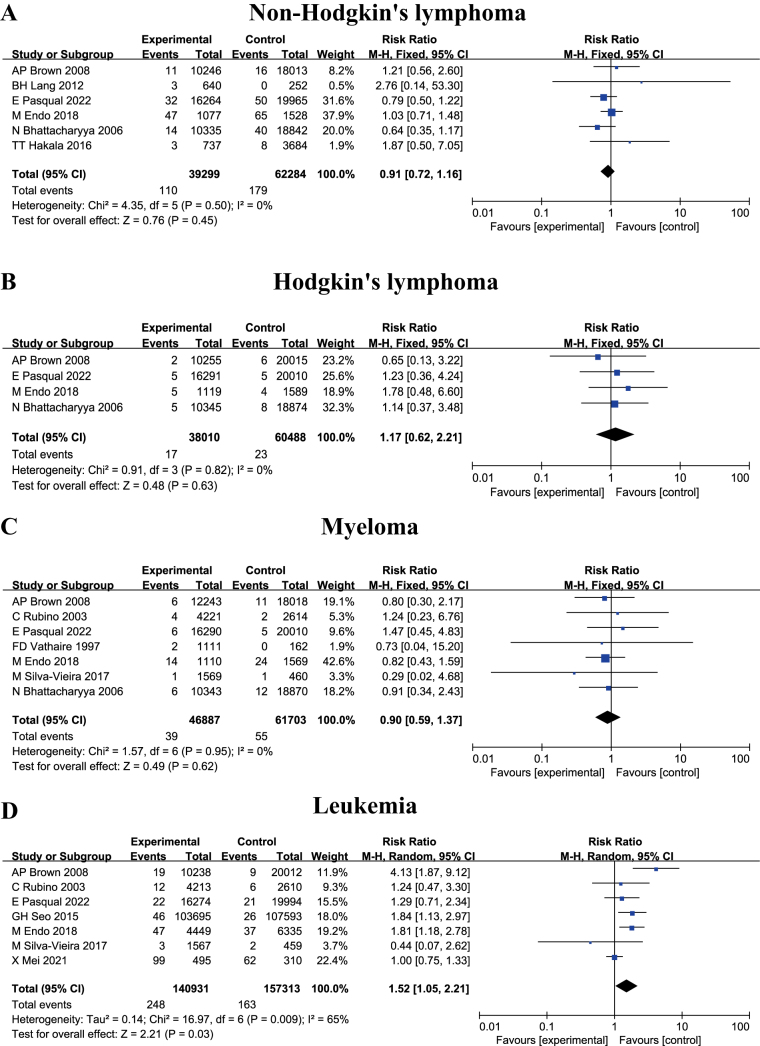
Forest plots depicting RR of SHMs in DTC patients administered RAI versus those not exposed, stratified by reported SHM subtypes: (A) non-Hodgkin’s lymphoma; (B) Hodgkin’s lymphoma; (C) Myeloma; (D) Leukemia.

To enable a more granular assessment of subtype-specific associations, SHMs were categorized into non-Hodgkin’s lymphoma, Hodgkin’s lymphoma, myeloma, and leukemia. The stratified analysis yielded the following RRs: non-Hodgkin’s lymphoma, 0.91 (95% CI: 0.72–1.16, *P* = 0.45); Hodgkin’s lymphoma, 1.17 (95% CI: 0.62–2.21, *P* = 0.63); myeloma, 0.90 (95% CI: 0.59–1.37, *P* = 0.62); and leukemia, 1.52 (95% CI: 1.05–2.21, *P* = 0.03), as illustrated in Fig 4. Comparative risk estimates indicate elevated susceptibility to Hodgkin’s lymphoma and leukemia following thyroid cancer surgery with RAI treatment relative to non-RAI cohorts, with leukemia exhibiting the highest risk and reaching statistical significance.

### Risk of SHMS in RAI dose

**Figure 5.**
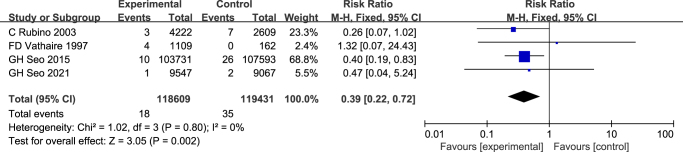
Forest plot illustrating RR of SHMs in DTC patients undergoing RAI therapy versus those untreated, based on studies with reported RAI doses <3.7 GBq.

Prior studies have indicated a dose-dependent association between RAI and SHMs. To further assess this relationship, subgroup analysis was conducted based on available data from multiple investigations detailing RAI dosage (Fig. 5). When the administered dose was <3.7 GBq, the pooled RR was 0.39 (95% CI: 0.22–0.72, *P* = 0.002), with no observed heterogeneity among studies (*P* = 0.80, *I*^2^ = 0%), as analyzed under a fixed-effects model. These results suggest that RAI doses below 3.7 GBq do not confer an elevated risk of SHMs.

## Discussion

DTC represents a well-characterized histopathological subtype of follicular cell-derived thyroid carcinoma. Its indolent biological behavior and limited invasiveness contribute to a 10-year survival rate exceeding 90% among affected individuals^[36]^. RAI has been widely employed as an adjuvant treatment for thyroid cancer for over five decades. Nonetheless, the association between RAI exposure and elevated SPM risk in thyroid cancer survivors remains controversial^[37]^. Although prior studies have reported a potential link between RAI and SHMs, the evidence is inconsistent. Methodological limitations – particularly inadequate follow-up duration and small sample sizes – may partially account for the observed discrepancies. In addition, prior investigations predominantly examined the association between RAI and overall SPMs without isolating SHMs as a distinct outcome, potentially introducing misclassification bias. This meta-analysis incorporates data from 17 studies to systematically assess the relationship between RAI administration and subsequent SHM development. Results indicate a statistically significant elevation in SHM risk among DTC patients who underwent RAI therapy compared to those who did not, particularly with extended follow-up periods.

Limited evidence suggests an association between RAI and the development of SHMs^[38,39]^. Current consensus indicates that RAI yields minimal therapeutic advantage in low-risk patients, while its role in reducing recurrence or mortality among moderate-risk individuals remains controversial. The decision to administer RAI should be based on a comprehensive risk-benefit evaluation^[40]^. A retrospective cohort study conducted in Korea involving 147 674 patients treated with RAI between 2004 and 2018 identified a dose-dependent increase in SHMs incidence, with hazard ratios of 1.214 for 101–200 mCi, 1.422 for 201–300 mCi, and 1.693 for doses exceeding 300 mCi^[41]^. In contrast, analysis by Rubino *et al* found that external radiotherapy, when treated as a binary variable (exposed vs. unexposed), did not significantly influence the relationship between RAI dose and SHMs risk^[23]^. This conclusion was based on pooled data from 17 studies including 586 260 patients with follow-up durations ranging from 2.4 to 20 years. The meta-analysis identified a dose-related association between RAI and SHMs. Furthermore, subgroup analysis revealed a significant increase in leukemia risk following RAI exposure, whereas no such association was observed when the RAI dose was below 3.7 GBq.

Subgroup analysis by SHM subtype indicated elevated RRs for Hodgkin lymphoma and leukemia among RAI-treated patients compared to untreated counterparts, with a statistically significant increase observed specifically for leukemia. The precise biological mechanism through which RAI preferentially induces leukemia, as opposed to other hematologic malignancies, remains undefined. Prior research has implicated RAI as a β-ray emitter potentially linked to leukemogenesis^[42,43]^. Additional studies suggest that ionizing radiation may contribute to leukemia development through induction of DNA double-strand breaks and other genotoxic pathways^[44]^. The risk of SHMs appears to exhibit dose dependency. Among the 17 studies reviewed, five identified a threshold of 3.7 GBq, one proposed 18.5 GBq, and two cited 1.11 GBq as relevant cut-off points. However, insufficient data precluded inclusion of the 18.5 and 1.11 GBq thresholds in the meta-analysis. Consequently, 3.7 GBq was selected as the reference value for evaluating dose–response relationships. Subgroup analysis by dosage revealed that RAI administration below 3.7 GBq was associated with a significantly lower RR compared to no RAI treatment, suggesting no increased SHM risk at this dosage level. Advanced tumor stage is typically correlated with the use of higher and more frequent RAI doses. Of the 17 studies analyzed, 7 provided thyroid cancer staging data, yet only one explored its association with SHM risk^[34]^. In that study, Mei *et al* reported no statistically significant relationship between higher tumor staging and SHM incidence. Additional investigations are required to clarify whether disease stage contributes to SHM susceptibility.

Several limitations merit consideration. First, potential screening bias in studies assessing SPM incidence may affect reliability, as patients with a cancer diagnosis typically undergo more intensive surveillance, increasing the likelihood of secondary malignancy detection. Second, the included 17 studies exhibit considerable heterogeneity in follow-up duration, ranging from 2.4 to 35 years. Disparities in follow-up length and methodology could alter the probability of identifying secondary tumors, thereby introducing variability and potential bias.

Third, temporal factors and evolving clinical practices may influence interpretation. Prior to the 2015 revision of ATA guidelines, pediatric thyroid cancer patients frequently receive iodine-131 doses comparable to those administered to adults, resulting in higher cumulative radiation exposure and possibly elevating the risk of secondary primary tumor development^[45]^. Additionally, improvements in medical care and increased post-surgical survival over time may extend the window during which secondary tumors emerge, further affecting incidence rates.

## Conclusion

The present meta-analysis indicates a potential association between RAI therapy and an elevated risk of SHMs in patients with DTC, with leukemia emerging as the most consistently implicated subtype. However, no statistically significant correlation was observed for other SHMs. At RAI doses below 3.7 GBq, the risk does not appear to increase appreciably. Caution is warranted in clinical decision-making regarding RAI administration. Validation through large-scale, prospective, multicenter studies remains essential to substantiate these observations.

## Data Availability

This manuscript constitutes original and valid research. No portion of its content has been published previously, nor is it under concurrent review elsewhere by the same authorship.

## Research registration unique identifying number (UIN)

The PROSPERO database (http://www.crd.york.ac.uk/PROSPERO/) logs the protocol for this meta-analysis under registration number 518211.

## References

[1] Wang Z Ji X Zhang H. Clinical and molecular features of progressive papillary thyroid microcarcinoma. Int J Surg 2024;110:2313–22. 38241301 10.1097/JS9.0000000000001117 PMC11019976

[2] Han B Zheng R Zeng H. Cancer incidence and mortality in China, 2022. J Natl Cancer Cent 2024;4:47–53. 39036382 10.1016/j.jncc.2024.01.006 PMC11256708

[3] Haugen BR Alexander EK Bible KC. 2015 American thyroid association management guidelines for adult patients with thyroid nodules and differentiated thyroid cancer: the American thyroid association guidelines task force on thyroid nodules and differentiated thyroid cancer. Thyroid 2016;26:1–133. 26462967 10.1089/thy.2015.0020 PMC4739132

[4] Hebestreit H Biko J Drozd V. Pulmonary fibrosis in youth treated with radioiodine for juvenile thyroid cancer and lung metastases after Chernobyl. Eur J Nucl Med Mol Imaging 2011;38:1683–90. 21626048 10.1007/s00259-011-1841-x

[5] Romanenko AY Finch SC Hatch M. The Ukrainian-American study of leukemia and related disorders among Chernobyl cleanup workers from Ukraine: III. Radiation risks. Radiat Res 2008;170:711–20. 19138038 10.1667/RR1404.1 PMC2856603

[6] Molenaar RJ Sidana S Radivoyevitch T. Risk of hematologic malignancies after radioiodine treatment of well-differentiated thyroid cancer. J Clin Oncol 2018;36:1831–39. 29252123 10.1200/JCO.2017.75.0232 PMC8462524

[7] Seo GH Cho YY Chung JH. Increased risk of leukemia after radioactive iodine therapy in patients with thyroid cancer: a nationwide, population-based study in Korea. Thyroid 2015;25:927–34. 26133388 10.1089/thy.2014.0557

[8] Tulchinsky M Binse I Campennì A. Radioactive iodine therapy for differentiated thyroid cancer: lessons from confronting controversial literature on risks for secondary malignancy. J Nucl Med 2018;59:723–25. 29653977 10.2967/jnumed.118.211359

[9] Cappagli V Caldarella A Manneschi G. Nonthyroidal second primary malignancies in differentiated thyroid cancer patients: is the incidence increased comparing to the general population and could it be a radioiodine therapy consequence? Int J Cancer 2020;147:2838–46. 32449158 10.1002/ijc.33116

[10] Marti JL Jain KS Morris LG. Increased risk of second primary malignancy in pediatric and young adult patients treated with radioactive iodine for differentiated thyroid cancer. Thyroid 2015;25:681–87. 25851829 10.1089/thy.2015.0067 PMC4948196

[11] Agha RA Mathew G Rashid R. Transparency In The reporting of Artificial INtelligence – the TITAN guideline. Premier J Sci 2025;10:100082.

[12] Moher D Liberati A Tetzlaff J. Preferred reporting items for systematic reviews and meta-analyses: the PRISMA statement. PLoS Med 2009;6:e1000097. 19621072 10.1371/journal.pmed.1000097 PMC2707599

[13] Kussainova A Kassym L Akhmetova A. Vitiligo and anxiety: a systematic review and meta-analysis. PLoS One 2020;15:e0241445. 33170870 10.1371/journal.pone.0241445 PMC7654800

[14] He X Wang P Wang Z. Thyroid antibodies and risk of preterm delivery: a meta-analysis of prospective cohort studies. Eur J Endocrinol 2012;167:455–64. 22826476 10.1530/EJE-12-0379

[15] Thangaratinam S Tan A Knox E. Association between thyroid autoantibodies and miscarriage and preterm birth: meta-analysis of evidence. BMJ 2011;342:d2616. 21558126 10.1136/bmj.d2616 PMC3089879

[16] Higgins JP Thompson SG. Quantifying heterogeneity in a meta-analysis. Stat Med 2002;21:1539–58. 12111919 10.1002/sim.1186

[17] Megens MR Churilov L Thijs V. New-onset atrial fibrillation after coronary artery bypass graft and long-term risk of stroke: a meta-analysis. J Am Heart Assoc 2017;6:e007558. 29273637 10.1161/JAHA.117.007558 PMC5779055

[18] Borenstein M Hedges LV Higgins JP. A basic introduction to fixed-effect and random-effects models for meta-analysis. Res Synth Methods 2010;1:97–111. 26061376 10.1002/jrsm.12

[19] Stuck AE Rubenstein LZ Wieland D. Bias in meta-analysis detected by a simple, graphical test. Asymmetry detected in funnel plot was probably due to true heterogeneity. BMJ 1998;316:469. PMC2665578 9492685

[20] Egger M Davey Smith G Schneider M. Bias in meta-analysis detected by a simple, graphical test. BMJ 1997;315:629–34. 9310563 10.1136/bmj.315.7109.629 PMC2127453

[21] Shea BJ Reeves BC Wells G. AMSTAR 2: a critical appraisal tool for systematic reviews that include randomised or non-randomised studies of healthcare interventions, or both. BMJ 2017;358:j4008. 28935701 10.1136/bmj.j4008 PMC5833365

[22] de Vathaire F Schlumberger M Delisle MJ. Leukaemias and cancers following iodine-131 administration for thyroid cancer. Br J Cancer 1997;75:734–39. 9043033 10.1038/bjc.1997.130 PMC2063327

[23] Rubino C de Vathaire F Dottorini ME. Second primary malignancies in thyroid cancer patients. Br J Cancer 2003;89:1638–44. 14583762 10.1038/sj.bjc.6601319 PMC2394426

[24] Bhattacharyya N Chien W. Risk of second primary malignancy after radioactive iodine treatment for differentiated thyroid carcinoma. Ann Otol Rhinol Laryngol 2006;115:607–10. 16944659 10.1177/000348940611500806

[25] Brown AP Chen J Hitchcock YJ. The risk of second primary malignancies up to three decades after the treatment of differentiated thyroid cancer. J Clin Endocrinol Metab 2008;93:504–15. 18029468 10.1210/jc.2007-1154

[26] Lang BHH Wong IOL Wong KP. Risk of second primary malignancy in differentiated thyroid carcinoma treated with radioactive iodine therapy. Surgery 2012;151:844–50. 22341041 10.1016/j.surg.2011.12.019

[27] Al-Qahtani KH Al-Asiri M Tunio MA. Prevalence and treatment outcomes of second primary malignancies in Saudi patients with differentiated thyroid cancers. Saudi Med J 2015;36:442–48. 25828281 10.15537/smj.2015.4.10341 PMC4404478

[28] Ko KY Kao CH Lin CL. (131)I treatment for thyroid cancer and the risk of developing salivary and lacrimal gland dysfunction and a second primary malignancy: a nationwide population-based cohort study. Eur J Nucl Med Mol Imaging 2015;42:1172–78. 25900274 10.1007/s00259-015-3055-0

[29] Hakala TT Sand JA Jukkola A. Increased risk of certain second primary malignancies in patients treated for well-differentiated thyroid cancer. Int J Clin Oncol 2016;21:231–39. 26410770 10.1007/s10147-015-0904-6

[30] Hirsch D Shohat T Gorshtein A. Incidence of nonthyroidal primary malignancy and the association with (131)I treatment in patients with differentiated thyroid cancer. Thyroid 2016;26:1110–16. 27302111 10.1089/thy.2016.0037

[31] Silva-Vieira M Carrilho Vaz S Esteves S. Second primary cancer in patients with differentiated thyroid cancer: does radioiodine play a role? Thyroid 2017;27:1068–76. 28614983 10.1089/thy.2016.0655

[32] Endo M Liu JB Dougan M. Incidence of second malignancy in patients with papillary thyroid cancer from surveillance, epidemiology, and end results 13 dataset. J Thyroid Res 2018;2018:8765369. 30046434 10.1155/2018/8765369 PMC6038658

[33] Mei X Yao X Feng F. Risk and outcome of subsequent malignancies after radioactive iodine treatment in differentiated thyroid cancer patients. BMC Cancer 2021;21:543. 33980182 10.1186/s12885-021-08292-8 PMC8117631

[34] Seo GH Kong KA Kim BS. Radioactive iodine treatment for children and young adults with thyroid cancer in South Korea: a population-based study. J Clin Endocrinol Metab 2021;106:e2580–e2588. 33755732 10.1210/clinem/dgab192

[35] Pasqual E Schonfeld S Morton LM. Association between radioactive iodine treatment for pediatric and young adulthood differentiated thyroid cancer and risk of second primary malignancies. J Clin Oncol 2022;40:1439–49. 35044839 10.1200/JCO.21.01841 PMC9061144

[36] Hundahl SA Fleming ID Fremgen AM. A National Cancer Data Base report on 53,856 cases of thyroid carcinoma treated in the U.S., 1985-1995. Cancer 1998;83:2638–48. 9874472 10.1002/(sici)1097-0142(19981215)83:12<2638::aid-cncr31>3.0.co;2-1

[37] Van Nostrand D. The benefits and risks of I-131 therapy in patients with well-differentiated thyroid cancer. Thyroid 2009;19:1381–91. 20001720 10.1089/thy.2009.1611

[38] Kim KJ Kim KJ Choi J. Linear association between radioactive iodine dose and second primary malignancy risk in thyroid cancer. J Natl Cancer Inst 2023;115:695–702. 36821433 10.1093/jnci/djad040 PMC10248848

[39] Kim M Kim H Park S. Risk factors for second primary malignancies following thyroid cancer: a nationwide cohort study. Eur J Endocrinol 2022;186:561–71. 35286279 10.1530/EJE-21-1208

[40] Iyer NG Morris LG Tuttle RM. Rising incidence of second cancers in patients with low-risk (T1N0) thyroid cancer who receive radioactive iodine therapy. Cancer 2011;117:4439–46. 21432843 10.1002/cncr.26070 PMC3155861

[41] Hong CM Son J Hyun MK. Second primary malignancy after radioiodine therapy in thyroid cancer patient: a nationwide study. Nucl Med Mol Imaging 2023;57:275–86. 37982105 10.1007/s13139-023-00818-1 PMC10654320

[42] Kendall GM Little MP Wakeford R. A record-based case-control study of natural background radiation and the incidence of childhood leukaemia and other cancers in Great Britain during 1980-2006. Leukemia 2013;27:3–9. 22766784 10.1038/leu.2012.151 PMC3998763

[43] Henshaw DL Eatough JP Richardson RB. Radon as a causative factor in induction of myeloid leukaemia and other cancers. Lancet 1990;335:1008–12. 1970069 10.1016/0140-6736(90)91071-h

[44] Dainiak N. Hematologic consequences of exposure to ionizing radiation. Exp Hematol 2002;30:513–28. 12063018 10.1016/s0301-472x(02)00802-0

[45] Palaniappan R Krishnamurthy A Rajaraman SS. Management outcomes of pediatric and adolescent papillary thyroid cancers with a brief review of literature. Indian J Cancer 2018;55:105–10. 30147104 10.4103/ijc.IJC_486_17

